# Treating perfectionism using internet-based cognitive behavior therapy: A study protocol for a randomized controlled trial comparing two types of treatment

**DOI:** 10.1016/j.invent.2020.100338

**Published:** 2020-08-27

**Authors:** Monica Buhrman, Olle Gelberg, Filip Jovicic, Katarina Molin, David Forsström, Gerhard Andersson, Per Carlbring, Roz Shafran, Alexander Rozental

**Affiliations:** a Department of Psychology, Uppsala University, Sweden; b Department of Clinical Neuroscience, Karolinska Institutet, Sweden; c Department of Psychology, Stockholm University, Sweden; d Department of Behavioural Sciences and Learning, Linköping University, Sweden; e UCL Great Ormond Street Institute of Child Health, University College London, UK

**Keywords:** Perfectionism, Cognitive behavior therapy, Unified protocol, Internet-based, Randomized controlled trial, Study protocol

## Abstract

Perfectionism is characterized by setting high standards and striving for achievement, sometimes at the expense of social relationships and wellbeing. Despite sometimes being viewed as a positive feature by others, people with perfectionism tend to be overly concerned about their performance and how they are being perceived by people around them. This tends to create inflexible standards, cognitive biases, and performance-related behaviors that maintain a belief that self-worth is linked to accomplishments. Cognitive behavior therapy has been shown to be a viable treatment for perfectionism, both in terms of reducing levels of perfectionism and improving psychiatric symptoms. Furthermore, a number of recent studies indicate that it can be successfully delivered via the Internet, both with regular support and guidance on demand from a therapist. In the present study protocol, a clinical trial for perfectionism is described and outlined. In total, 128 participants will be recruited and randomized to either a treatment that has already been demonstrated to have many benefits, Internet-based Cognitive Behavior Therapy for perfectionism (iCBT-P), or an active comparison condition, Internet-based Unified Protocol (iUP), targeting the emotions underlying depression and anxiety disorders. The results will be investigated with regard to self-reported outcomes of perfectionism, psychiatric symptoms, self-compassion, and quality of life, at post-treatment and at six- and 12-month follow-up. Both iCBT-P and iUP are expected to have a positive impact, but the difference between the two conditions in terms of their specific effects and adherence are currently unknown and will be explored. The clinical trial is believed to lead to a better understanding of how perfectionism can be treated and the specificity of different treatments.

## Highlights

• A randomized controlled trial for perfectionism will be performed.
• Cognitive Behavior Therapy will be compared to Unified Protocol.
• Effects on self-rated perfectionism and psychiatric symptoms will be explored.
• Guidance on demand will be provided.
• Follow-up at six and 12 months will be made to demonstrate the long-term effects.

## 1 Introduction

Perfectionism refers to the disposition to set high standards and strive for achievement, typically characterized by the refusal to accept actions and results that fall short of perfection. According to Shafran, Cooper, and Fairburn (2002, p. 778), perfectionism is characterized by *“the overdependence of self-evaluation on the determined pursuit of personally demanding, self-imposed standards in at least one highly salient domain, despite adverse consequences”*. As such, people with perfectionism tend to link their sense of self-worth to the realization of subjectively imposed far-reaching goals, making them prone to self-criticism and negative self-evaluation when these are not met. It is believed that at the core of perfectionism lies highly rigid demands that set the occasion for inflexible rules and intermediate beliefs, e.g., “I cannot make mistakes” and “If I do not succeed at this, I am utterly useless”. This will in turn generate various cognitive biases and performance-related behaviors that maintain the need for perfection, for example, dichotomous thinking (i.e., seeing outcomes as either black or white), selective attention (disregarding the positives), and repeated checking and seeking reassurance from others (Shafran et al., 2016).

Because perfectionism is considered to have trait-like features it is not regarded as a condition with clear boundaries between functional and dysfunctional distributions. Hence, cutoffs differentiating normal and pathological perfectionism are difficult to establish, which makes it hard to estimate the prevalence rate of those in need of treatment. However, empirical evidence demonstrates that perfectionism can lead to a number of detrimental consequences and is elevated among patients with psychiatric disorders (Egan et al., 2011). Perfectionism is for instance associated with a persistent fear of failure and negative evaluation and various emotional, social, physical, cognitive, and behavioral implications (Stoeber, 2017). A systematic review and meta-analysis by Limburg et al. (2017) of 284 studies also examined the relationship between perfectionism and psychopathology. In line with the present understanding of perfectionism, the findings separate the two higher-order dimensions that constitute the construct. First, *perfectionistic strivings*, the propensity to set high standards and being demanding of yourself. Second, *perfectionistic concerns*, the inclination of being self-critical in terms of your own actions, being preoccupied by how others perceive yourself, and having difficulties feeling content about your performance (Stoeber et al., 2020). Although both dimensions are correlated with psychiatric symptoms, perfectionistic strivings seem to be primarily linked to eating disorders (*r* = .36 for bulimia nervosa and .56 for anorexia nervosa). Meanwhile, perfectionistic concerns were mainly related to general psychological distress (*r* = .42), depression (*r* = .40), and anxiety disorders (*r* = .30), potentially revealing distinctive clinical profiles (Limburg et al., 2017). In a recent study of possible mediators, Smith et al. (2020) revealed two reasons for this negative impact. One being due to increased stress, as perfectionistic individuals engage in more stressful activities overall and interpret minor setbacks as failures. The second being cause by so-called social disconnection, that is, the tendency to interpret social interactions as being characterized by criticism, which can lead to isolation and focus on achievement at the expense of closer social relationships (Chen et al., 2015).

### 1.1 Treating perfectionism

Perfectionism is regarded as a transdiagnostic phenomenon that can be problematic in itself, but that is also involved in processes that can create or maintain other psychiatric symptoms. This notion is confirmed by the fact that perfectionism is elevated in many conditions, that it predicts the onset and recovery from depression, and that it has strong links to, for example, obsessive-compulsive disorder and social anxiety disorder (Egan et al., 2011). Perfectionism can also affect the course of treatment for these conditions by leading to poorer therapeutic alliance, non-response, and drop-out, particularly with regard to the engagement with certain interventions, such as exposure to fearful stimuli or using behavioral activation (Egan et al., 2016). Hence, there are several reasons to be aware of perfectionism among patients as it can affect both adherence to treatment and result in worse prognostic outlook and increase the risk of relapse. For this reason, Shafran et al. (2002) proposed a cognitive-behavioral conceptualization of perfectionism that can help guide therapists in their assessment and treatment. This stems from their definition of perfectionism being characterized by tightly held inflexible standards, and intends to target cognitive biases and performance-related behaviors, such as via behavioral experiments. Four components were considered important: 1) providing psychoeducation about perfectionism and creating an individualized conceptualization 2) broadening the domains for self-evaluation 3) testing out beliefs and predictions, and 4) addressing personal standards and self-criticism. The treatment has been developed into a manual (Egan et al., 2016), and a self-help book (Shafran et al., 2018), which can be used either stand-alone or in relation to other evidence-based interventions when perfectionism is relevant to address.

To date, the above-mentioned treatment has been evaluated in a series of clinical trials and different settings and formats, such as one-on-one face-to-face, in groups, and unguided self-help. A systematic review and meta-analysis of cognitive behavior therapy involving eight studies demonstrated moderate to large within-group effect sizes Hedge's *g* of 0.79, 95% Confidence Interval (CI) [0.44, 1.14] and 1.33, 95% CI [1.02, 1.64] for self-reported outcomes of perfectionism, while simultaneously having a moderate positive impact on anxiety (.52), 95% CI [0.23, 0.81], and depression (.64), 95% CI [0.35, 0.92] (Lloyd et al., 2015). Recently, several examples of administering the treatment via the Internet have also been evaluated with promising results. In these studies, the outline has been mirrored but delivered as guided self-help programs of 8–12 weeks, demonstrating intention-to-treat between-group effect sizes of Cohen's *d* of 1.01, 95% CI [0.63, 1.39] for perfectionistic concerns, and .67, 95% CI [0.31, 1.04] for perfectionistic strivings (Shafran et al., 2017), and 1.00, 95% CI [0.66, 1.33], and .68 [0.36, 1.00], respectively in Rozental et al. (2017), with maintained benefits at both six and 12 months (Rozental et al., 2018). One study also examined the use of guidance-on-demand with similar results, 1.00, 95% CI [0.51, 1.47], and .72, 95% CI [0.24, 1.18] (Zetterberg et al., 2019). In another systematic review and meta-analysis consisting of 10 studies it was also observed that such a format is comparable to seeing a therapist individually face-to-face (Suh et al., 2019). Thus, it can be suggested that cognitive behavior therapy delivered via the Internet can be helpful in overcoming perfectionism and related psychiatric symptoms. Internet-based treatments are generally seen as a viable option for various conditions, e.g., depression, anxiety disorders, and psychosomatic issues, with similar outcomes as other means of delivery, with the advantage of being available regardless of geographical location (Andersson, 2018). Given that perfectionism might not always be recognized as something that warrants clinical attention in routine care, and due to the lack of trained therapists, Internet-based cognitive behavior therapy could help disseminate an effective treatment for this type of problem (Andersson et al., 2019).

### 1.2 Study protocol for a randomized controlled trial

So far, cognitive behavior therapy has been shown to produce outcomes whereby perfectionistic individuals are able to manage the difficulties associated with having to unremittingly strive for achievement and attaining certain standards. This has been confirmed not only by reduced levels of self-reported perfectionism (Lloyd et al., 2015; Suh et al., 2019), but also qualitative investigations demonstrating that patients learn how to deal with situations differently, become better at shifting focus from performance, and broaden the domains for self-evaluation (Rozental et al., 2020). In addition, preliminary evidence implies that psychiatric symptoms can be targeted using cognitive behavior therapy for perfectionism despite not being specifically addressed (Kothari et al., 2019). However, additional studies are needed in order to replicate the results. In particular, comparing two forms of treatment is important to get a better idea of the true effects given that an inactive comparator, e.g., waiting list, can yield inflated results (Cuijpers and Cristea, 2016). In Suh et al. (2019), only half of the 10 clinical trials included in their systematic review and meta-analysis included some form of treatment, which mostly consisted of providing participants with a self-help book. Hence, in the present study protocol, the comparison between two forms of cognitive behavior therapy will be described and outlined; the manual developed by Egan et al. (2016), here referred to as Internet-based Cognitive Behavior Therapy for Perfectionism (iCBT-P), which has been tested on several occasions, and Internet-based Unified Protocol (iUP), a transdiagnostic manual that intends to target the emotional aspects underlying the symptoms involved in depression and anxiety disorders (Ellard et al., 2010). Studies this approach have demonstrated promising results when examined in itself and when compared to treatments that are diagnosis-specific (Barlow et al., 2017; Bullis et al., 2014; Farchione et al., 2012). As to date, Unified Protocol has not been tested for perfectionism, but given its focus on the emotions involved in many conditions, it is reasonable to think that it will have a positive impact on the emotional responses that accompany perfectionism. Moreover, given that iCBT-P and iUP were originally developed for different purposes, that is, perfectionism versus psychiatric symptoms, a clinical trial makes it possible to explore the specificity of the two treatments.

The clinical trial will thus test two treatments for perfectionism over eight weeks, and will be delivered to self-referred participants who are recruited on the basis of experiencing perfectionism as a major concern. Both will be administered via the Internet in order to replicate prior studies of the same mean of delivery. Given that no investigation has previously tested the effects of iUP for perfectionism, no hypothesis regarding, for instance, superiority is possible to provide. However, because iCBT-P was developed specifically for targeting perfectionism, it is assumed to have a greater impact on this particular issue. The clinical trial will involve the following research questions; 1) what are the effects on self-rated perfectionism and psychiatric symptoms of an eight-week Internet-delivered treatment with guidance on demand (iCBT-P)? 2) what are the effects on the same outcomes for iUP? 3) to what extent has treatment affected other domains, such as relationships, as measured using subjective ratings? 4) how are the treatments experienced by the participants themselves, as explored using qualitative interviews?

## 2 Material and methods

### 2.1 Participants and procedure

Participants will be recruited via advertisements and social media. Those who identify themselves as perfectionists will be referred to a website that has been created specifically for the purpose of the clinical trial. This mean of recruitment has effectively been used before for issues that are not considered to be diagnoses (Rozental et al., 2017), and has the advantage of reaching individuals who themselves regard perfectionism as problematic. The website will consist of general information about the study, the researchers and therapists, ethical issues, and the conditions for their participation. Once an individual signs up to participate, she enters a secure platform using an autogenerated username, e.g., 1234abcd, strong personal password, and six-letter code sent out as a SMS (for more details, see Vlaescu et al., 2016). This type of authorization process is used by many authorities and is regarded to be safe for sharing sensitive data. When the individual is logged on, she then completes a number of questions of demographic nature and all of the self-report measures listed below. These are used to determine eligibility to be included. Those who meet inclusion criteria are later interviewed via telephone using the Mini-International Neuropsychiatric Interview version 7.0 to confirm diagnoses and screen for conditions that are considered a reason for exclusion (MINI; Lecrubier et al., 1997).

**Fig. 1:**
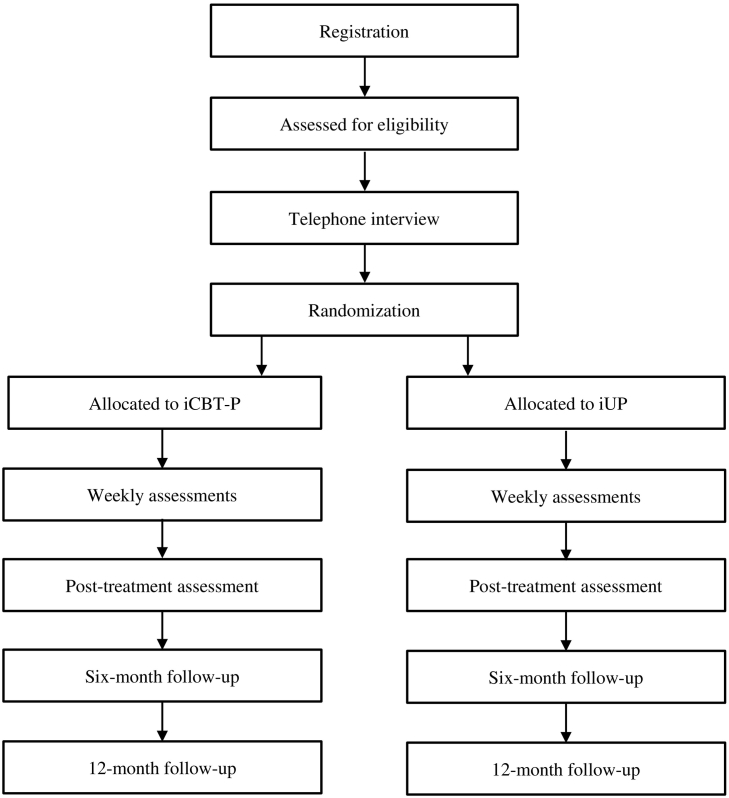
Flow chart for the clinical trial.

Following recruitment, all included participants are randomized by an independent individual not involved in the study. Randomization will be done to the two conditions according to a 1:1 ratio and using a random numbers generator (www.random.org). Treatment content, communication with therapists, technical support, and all self-report measures can be accessible through the secure platform, using the same type of login as described above. Participants will connect their own emails to their autogenerated username, however only notices to log on to the secure platform will be sent out. The treatments are both eight weeks long, after which the participants complete the same type of self-report measures as before (two of them will also be filled out weekly throughout treatment), with a similar procedure being applied at six and 12 months following treatment completion, see Fig. 1 for an overview.

Should the status of a participant deteriorate, as identified using weekly measures with the Patient Health Questionnaire (PHQ-9), the secure platform will notify her assigned therapist about the matter, who then contacts the individual for further help or referral. Unless vital to refer to another healthcare provider, the participant can remain in treatment. The principal investigators (MB and AR) will assume overall responsibility for clinical issues concerning the participants.

### 2.2 Inclusion and exclusion criteria

Individuals interested in participating in the clinical trial are self-referred on the basis of recognizing perfectionism as an ongoing concern. In order to determine eligibility, a set of inclusion and exclusion criteria will be applied; 1) ≥18 years of age 2) adequate reading and writing levels in Swedish 3) a computer, smartphone, or tablet with Internet access, and 4) elevated levels of perfectionism, as determined by a score of >29 on the subscale Concerns over Mistakes on the Frost Multidimensional Perfectionism Scale (FMPS; Frost et al., 1990). Diagnoses are not a reason for exclusion unless they require more immediate care, e.g., anorexia nervosa, substance abuse, bipolar disorder, psychosis, and schizophrenia, as assessed during the telephone interview. Those who display severe depression or suicidality during screening will be contacted and referred to their local healthcare provider, as determined by a score of >15 or > 2 points on the item on suicidal ideation on the PHQ-9 (Kroenke et al., 2001). Excluded individuals will not be able to take part in treatment.

### 2.3 Withdrawal

Participants can withdraw from the clinical trial at any moment. The reason for this will be explored for research purposes, but the individual will be informed that she can defer from specifying her motive.

### 2.4 Self-report measures

The clinical trial will include two main outcomes concerning the levels of perfectionism among the participants; the Clinical Perfectionism Questionnaire (CPQ; Fairburn et al., 2003), and the FMPS (Frost et al., 1990). The CPQ consists of 12 items that are scored on a four-point Likert-scale 1–4 (“Not at all” to “All of the time”), with two items that are in reverse (items 2 and 8), and employing a time-frame of one month to increase its clinical usefulness. The CPQ was translated to Swedish in a previous clinical trial, demonstrating good convergent and discriminant validity when compared to self-report measures of perfectionism and psychiatric symptoms, as well as adequate internal consistency (α = .68) and test-retest reliability (*r* = .62) (McMahan et al., submitted). The FMPS is scored on a five-point Likert-scale 1–5 (“Strongly disagree” to “Strongly agree”), with a total of 35 items that cover the six separate subscales Concern over Mistakes, Personal Standards, Doubts about Action, Parental Expectations, Parental Criticism, and Organization. However, only the first two are usually of interest in clinical trials as the other subscales are considered less related to the two higher-order dimensions of perfectionism (Limburg et al., 2017). The subscales of the FMPS has adequate to excellent internal consistencies (.88 and .83) and exhibits convergent and discriminant validity when compared to self-report measures of perfectionism and psychiatric symptoms (Purdon et al., 1999). The FMPS was originally translated to Swedish by Lundh et al. (1994). Apart from completing the two main outcomes during screening and at post-treatment and follow-up, participants will also fill out the CPQ weekly during treatment, see Table 1 for an overview.

**Table 1:** Overview of the self-report measures.

Self-report measure	Assessment											
	Screening	1	2	3	4	5	6	7	8	Post	6 months	12 months
CPQ	X	X	X	X	X	X	X	X	X	X	X	X
FMPS	X									X	X	X
PHQ-9	X	X	X	X	X	X	X	X	X	X	X	X
GAD-7	X									X	X	X
BBQ	X									X	X	X
SCS-SF	X									X	X	X
PPS	X									X	X	X
PSS	X									X	X	X
Credibility			X									
Domains^a^	X									X	X	X
Goals^b^	X									X	X	X
NEQ										X		

CPQ = Clinical Perfectionism Questionnaire; FMPS = Frost Multidimensional Perfectionism Scale; PHQ-9 = Patient Health Questionnaire; GAD-7 = Generalized Anxiety Disorder; BBQ = Brunnsviken Brief Quality of Life Scale; SCS-SF = Self-Compassion Scale - Short Form; PPS = Pure Procrastination Scale; PSS = Perceived Stress Scale; NEQ = Negative Effects Questionnaire.

a Interests/leisure, work/studies, friendships/social life, community engagement/spirituality, family life/parenting, rest/sleep, love/intimate relationships, and physical activity/diet.

b Goal attainment, as set and defined by the participants.

A number of secondary outcomes will also be included. The nine-item PHQ-9 assesses the degree of depression and is scored on a four-point Likert-scale 0–3 (“Not at all” to “Nearly every day”). The PHQ-9 is often used as a screening tool for depressive symptoms, has been validated against other self-report measures and clinical interviews of depression, and has an excellent internal consistency (.89) (Kroenke et al., 2001). The seven-item Generalized Anxiety Disorder (GAD-7) determines the level of anxiety and is scored on a four-point Likert-scale 0–3 (“Not at all” to “Nearly every day”). The GAD-7 is often used for screening for symptoms of anxiety and worry, corresponds well with other similar self-report measures, and has exhibited excellent internal consistency (.92) (Spitzer et al., 2006). The 12-item Brunnsviken Brief Quality of Life (BBQ) explores the quality of life in six areas, e.g., leisure and learning, and level of importance, e.g., “my leisure time is important to me”, which are then multiplied with each other. The BBQ is scored on a four-point scale 1–4 (“Strongly disagree” to “Strongly agree”), with a range in scores of 0–96. The BBQ demonstrates good convergent and discriminant validity, good classification ability, and has an adequate internal consistency (.76) (Lindner et al., 2016). The 12-item Self-Compassion Scale - Short Form (SCS-SF) tests the degree of self-compassion and is scored on a five-point scale 1–5 (“Almost never” to “Almost all of the time”). The SCS-SF has been shown to correlate with self-report measures of psychiatric symptoms, and has a good internal consistency (.86) (Raes et al., 2011). The 12-item Pure Procrastination Scale (PPS) determines the level of procrastination and is scored on a five-point Likert-scale 1–5 (“Seldom, or do not agree” to “Very often, or totally agree). The PPS has been shown to have good convergent and discriminant validity, and internal consistency (.78) (Rozental et al., 2014b; Steel, 2010). The 14-item Perceived Stress Scale (PSS) evaluates the subjective experience of general stress in various situations and is scored on a five-point Likert-scale 0–4 (“Never” to “Very often”), with seven items being scored in reverse (items 4–7, 9–10, and 13). The PSS has been shown to have good internal consistency (.84–.86) as well as good convergent and discriminant validity (Cohen et al., 1983). Lastly, the 32-item Negative Effects Questionnaire (NEQ) will be used to probe for unwanted and adverse events that might arise during treatment, which is scored on a five-point Likert-scale 0–4 (“Not at all” to “Extreme”), and classifies the incidents as caused by the treatment or other circumstances. The NEQ has demonstrated excellent internal consistency (.95) and is mainly used to descriptively determine the occurrence and nature of possible negative effects (Rozental et al., 2016). All of the listed secondary outcomes have previously been translated to Swedish. Moreover, with the exception of the PHQ-9, which is completed weekly during treatment, and the NEQ, which is only filled out at post-treatment, all self-report measures are completed during screening and at post-treatment and follow-up.

Furthermore, the perceived integrity of iCBT-P and iUP will be explored using the five-item Credibility/Expectancy Questionnaire (Borkovec and Nau, 1972), which is scored on a 10-point Likert-scale 0–10 (e.g., “Not at all logical” to “Very logical”). It has exhibited good internal consistency (.86–.90), with the factor expectancy being correlated with outcomes of treatment (Devilly and Borkovec, 2000). In addition, the effects of treatment will also be explored using subjective rvatings of their impact on different domains. This will entail eight aspects of life that each participant rates on a 10-point Likert-scale 0–10 with regard to how perfectionism is affecting them negatively (“Not at all” to “Very much”): interests/leisure, work/studies, friendships/social life, community engagement/spirituality, family life/parenting, rest/sleep, love/intimate relationships, and physical activity/diet. Participants will also define their own goals of treatment and to what extent they have lived up to them, also a 10-point Likert-scale 0–10.

### 2.5 Treatments and therapists

The clinical trial will randomly assign participants into one of two conditions; 1) iCBT-P, which is the manual developed by Egan et al. (2016) specifically for perfectionism, administered via the Internet, or 2) iUP, which is a transdiagnostic manual intended to target the shared emotional aspects that underlie depression and anxiety disorders, but here adapted to be Internet-delivered. Both treatments will be delivered in a similar manner over eight weeks, consisting of reading material, graphis, audio and video, and a number of exercises to be completed on the secure platform. The key content and main interventions in iCBT-P has been described in the introduction, but broadly involves gaining and understanding of the factors that maintain perfectionism, experimenting with new ways of thinking and behaving, expanding the source for self-evaluation, and managing self-criticism (Shafran et al., 2016), see Table 2 for an overview. Meanwhile, iUP can be regarded as an emotion-focused treatment that intends to raise awareness of the aversive experiences that an individual has a heightened emotional sensitivity for, and which it then tries to alter or avoid (Ellard et al., 2010). Fundamental to iUP is therefore to register and become more aware of the emotions, cognitions, and physical sensations that occur in difficult situation, and to try out more adaptive ways of coping in these instances. Both iCBT-P and iUP relies on techniques that are commonly used in cognitive behavior therapy. However, one crucial difference is that the former emphasizes knowledge acquisition and refuting dysfunctional beliefs to a great degree, such as by testing out predictions in real life, while the latter leans more toward exposure to emotional stimuli and using mindfulness to shift perspectives in relation to thoughts that evoke strong emotions.

**Table 2:** Treatment content.

Week	iCBT-P	iUP
1	Understanding your perfectionism, e.g., what is unhelpful perfectionism, what are the pros and cons of perfectionism	Emotional symptoms, e.g., what are emotional symptoms, is this treatment for me, registering your experiences, finding your motivation, setting goals
2	Your own model, values, and motivation, e.g., creating an individual conceptualization on what maintains your perfectionism, cost-benefit analysis	Understanding your emotions, e.g., introduction to emotions, what is an emotion, monitor your emotions
3	Surveys and experiments, e.g., introduction to behavioral experiments, pleasurable activities	Emotional awareness, e.g., introduction to emotional awareness, practicing non-judgmental mindfulness, anchoring
4	Dealing with perfectionistic behaviors, e.g., dealing with avoidance and safety behaviors, procrastination, and problem-solving	Thoughts, e.g., what are cognitive judgments, automatic judgments, identifying automatic judgments, thinking errors, cognitive restructuring
5	New ways of thinking, e.g., introduction to cognitive bias, dichotomous thinking, rigidity, focusing on negatives, disregarding positives	Behaviors, e.g., introduction to emotional avoidance, strategies for emotional avoidance, emotional behaviors, preventing avoidance and emotional responses
6	Self-criticism and self-compassion, e.g., introduction to self-compassion, becoming aware of self-critical thinking, practicing compassionate thinking	Emotional exposure, e.g., exposure to emotional situations, imagery, exposure to bodily sensations
7	Self-worth, e.g., introduction to self-worth, becoming less focused on performance, connecting self-worth to values	Continued motional exposure
8	Maintain and continue positive change, e.g., maintaining progress, preventing and managing different setbacks, Q&A, your own plan forward	Planning ahead, e.g., repeating skills and dealing with emotions, determining your progress, becoming your own therapist, long-term goals, maintenance, managing setbacks

iCBT-P = Internet-Based Cognitive Behavior Therapy for Perfectionism: iUP = Internet-based Unified Protocol.

In order to fit with the issues often experienced in perfectionism, a number of slight modifications had to be made to iUP. First, throughout the manual, examples were made perfectionism-relevant from its original focus on depression and anxiety disorders. Second, one module revolves around interoceptive exposure to the physical sensations that often occur in panic disorder, e.g., spinning around on a chair to evoke dizziness. This was considered inappropriate for a study on perfectionism and was therefore removed entirely. The same was also true for a few sections that focused on intrusions and compulsions common in obsessive-compulsive disorder.

Both treatments will be delivered on the secure platform and follow eight separate modules that are delivered as one module per week. Upon randomization, participants are also assigned their own therapist who they can contact when needed, i.e., guidance on demand. This is done via the secure platform, and as soon as a response has been made the participants will be notified once they enter. A message is also sent to their emails, but only to inform that they have to log on to access the response (i.e., no sensitive details are managed outside the secure platform). This approach was used in a previous study (Zetterberg et al., 2019), and did not differ from receiving regular help from a therapist (i.e., scheduled check-ins and feedback twice per week) in terms of outcomes or the number of modules and exercises that were completed. The therapists will consist of students that are either about to complete the study program in psychology (masters degree) who have undergone three semesters of clinical training in cognitive behavior therapy, or the study program in psychotherapy (bachelors degree, i.e., an advanced continuation program focusing specifically on clinical training in cognitive behavior therapy). The therapists respond to questions and provide support on the content and exercises in treatment, but do so freely in their own words. The therapists in the clinical trial will however receive training and supervision from a specialist in iCBT-P and iUP, respectively. Guidance is recorded to keep track of the number of messages sent between the participants and their therapists.

### 2.6 Ethics and registration

The clinical trial has received its ethics approval by the Swedish Ethical Review Authority (Dnr: 2020-01868), and has been registered at ClinicalTrials.gov (NCT04459260). Participants will provide informed consent during the recruitment process and screening. Great consideration will also be made to monitor the condition of those undergoing treatment, i.e., deterioration will be flagged automatically by the secure platform and relevant measures will be taken to ensure further help is introduced (see details under statistical analysis). Meanwhile unwanted and adverse events will also be explored after treatment has ended.

### 2.7 Timeline

The clinical trial is planned to recruit participants in September 2020. The treatments are expected to be completed in November 2020, with the two follow-up assessments being administered in June and November 2021. A submitted manuscript with the final results is planned to be ready in February 2022.

### 2.8 Statistical analysis

In the literature, no prior estimates are available for a power calculation when it comes to what constitutes a reasonable between-group effect size in terms of perfectionism. However, it is reasonable to assume a moderate standardized difference when comparing iCBT-P and iUP on the CPQ in favor of the former. This is based on the assumption that iCBT-P was developed specifically to target perfectionism. Thus, using G*Power to determine the sample size required to detect a Cohen's *d* of 0.50 (α of .05 and β of 0.80) suggest that 64 participants per condition (*n* = 128) will be required for the study. As for the within-group effect size for each condition between screening and post-treatment, the power achieved under these circumstances reveal that it will be possible to detect an effect size *d* of 0.36. The prior study by Zetterberg et al. (2019) demonstrated an effect size *d* of 1.19 on the CPQ for participants receiving iCBT-P with guidance on demand, and > 0.49 for all of the secondary outcomes, which indicates that the current clinical trial should be able to detect a difference on each of the self-report measures.

In terms of the statistical analyses, differences between conditions and between completers and non-completers will be examined using *t*-tests and Pearson *χ*^2^-tests. Conditional changes over time on both the CPQ and PHQ-9 will be modeled using an intention-to-treat linear mixed-effect model using data from screening, weekly assessment, and post-treatment assessment, and employing random slops, unstructured covariance matrix, and full maximum likelihood estimation with 100 iterations. The other self-report measures have fewer data points and are therefore considered inappropriate for mixed-effects modeling (Hesser, 2015). These will therefore be analyzed using repeated measures analysis of variance and using multiple imputation to deal with missing data. Effect sizes *d* will be calculated to determine the standardized differences in means within and between conditions, and 95% Confidence Intervals will be reported. Clinically significant change, criterion A, will be used to identify the number of participants who have recovered in their perfectionism. As perfectionism is not a diagnosis, recovery will be defined as reaching a statistical criterion of two standard deviations of change from the mean in the direction of a functional distribution on the CPQ, and exceeding measurement error as defined by the reliable change index (Jacobson and Truax, 1991). The latter will also be utilized to determine the number of participants who have experienced non-response and reliable deterioration (Rozental et al., 2014a).

### 2.9 Qualitative interviews

Following the post-treatment assessment, participants will be asked to be part of a qualitative interview that intends to investigate their experiences of undergoing treatment and its impact. In total, 12 participants from each condition will be recruited (*n* = 24), based on their results: recovery, non-response, and deterioration, as defined above. The interviews will be conducted via telephone and follow a semi-structured and open format.

## 3 Discussion

The present study protocol has described and outlined clinical trial that will compare two forms of cognitive behavior therapy delivered via the Internet; iCBT-P, specifically developed for treating perfectionism, and iUP, a transdiagnostic treatment for the emotional aspects that are believed to be an underlying process involved in depression and anxiety disorders. Both of the treatments will be delivered over the course of eight weeks and with guidance on demand. The clinical trial will first and foremost replicate prior findings concerning the benefits of cognitive behavior therapy for perfectionism and psychiatric symptoms (Rozental et al., 2017; Shafran et al., 2017; Zetterberg et al., 2019), but it will also be the first study where this treatment is compared to an active comparison condition. In addition, the two treatments make it possible to study their specificity in terms of outcomes, that is, to what extent each seem to have a positive impact on perfectionism and psychiatric symptoms, respectively. Although the clinical trial is not designed to test, for instance, superiority, iCBT-P is nonetheless expected to have a greater impact on perfectionism given that it was developed specifically for its treatment. The results from the study will be able to provide information that can be used for future studies on this issue. Furthermore, the self-report measures being used will allow an exploration of the effects on not only levels of perfectionism, but also psychiatric symptoms, self-compassion, and quality of life up to 12 months following treatment. Likewise, using a qualitative interview will make it possible to investigate the experiences of undergoing treatment of perfectionism via the Internet and the impact it might have on the participants.

The clinical trial has several strengths that should be discussed. Both of the principal investigators (MB and AR) as well as all of the researchers involved have extensive experiences of conducting studies on Internet-based cognitive behavior therapy, and the secure platform has been used in a large number of clinical trials (Vlaescu et al., 2016). Thus, this will guarantee an efficient recruitment period and administration of the treatments and self-report measures. Both iCBT-P and iUP have also received empirical support for their use and should therefore produce benefits for the participants receiving them. In addition, weekly assessments of perfectionism and depression improves statistical power and allows more advanced statistical modeling. However, there are also a number of limitations that should be addressed. Because perfectionism is not considered a diagnosis, determining who is eligible for treatment and who has recovered are complicated issues. Using a statistical criterion like the one that has been proposed is not uncommon in the absence of clear cutoffs, e.g., procrastination (Rozental et al., 2015). Nonetheless, other methods should be used to assess whether someone can be included and if recovery has in fact occurred. The clinical trial will therefore incorporate a number of additional ratings that can help to examine this, such as to what extent the participants think their perfectionism is affecting them negatively on various dimensions prior to initiating and after treatment, e.g., friendships/social life, and whether the goals of treatment has been accomplished or not. Qualitative interviews will also be conducted to explore the participants' experiences of the treatments and to what extent they have made an impact, similar to Rozental et al. (2020). Moreover, using self-referrals in clinical trials can be problematic as they do not always reflect the demographic composition of the general public. Whether this is true also for individuals having difficulties due to perfectionism is unclear, but should be explored by probing how they were recruited and to make an effort in advertising the study on multiple locations and through different media. Lastly, although adherence to treatment can be registered in terms of number of completed modules, determining how much time each participant spend on reading the content and doing the exercises is difficult to assess. The clinical trial will probe for this using open ended questions and self-reports, but a closer and more detailed inspection would have been preferred to explore dose-response issues.

## 4 Conclusion

The present study protocol has described a clinical trial on perfectionism that will help to increase the current understanding of how to treat a problem that often causes great distress to those afflicted. The aim is to provide more individuals with treatments that are efficacious and widely available. Although Internet-based cognitive behavior therapy may not suit everyone, it could become part of a stepped-care approach and first line of treatment for perfectionism that is accessible regardless of geographical location.
